# A Behavioral Characteristics Observational Measure of Youth with Somatic Symptom Disorder during Physical Rehabilitation

**DOI:** 10.3390/life13102078

**Published:** 2023-10-18

**Authors:** Sharon Barak, Jana Landa, Maya Gerner, Etzyona Eisenstein, Chen Arzoni Bardach, Tamar Silberg

**Affiliations:** 1 Department of Nursing, Faculty of Health Science, Ariel University, Ariel 4070000, Israel; 2 Department of Pediatric Rehabilitation, The Edmond and Lily Safra Children’s Hospital, Ramat-Gan 5262000, Israel; janna.landa@sheba.health.gov.il (J.L.); mayagerner@gmail.com (M.G.); etzyona.eisenstein@sheba.health.gov.il (E.E.); arzonit@gmail.com (C.A.B.); tamarsilberg@gmail.com (T.S.); 3 The Sackler School of Medicine, Tel Aviv University, P.O. Box 39040, Tel Aviv 6997801, Israel; 4 Department of Psychology, Bar-Ilan University, Ramat-Gan 5290002, Israel

**Keywords:** child somatization inventory, pain, rehabilitation, children, behavior

## Abstract

Background: Youth with somatic symptom disorder (SSD) present unique behavioral characteristics. Aims: To develop and examine the psychometric properties of an observational measure of behavioral characteristics for youth with SSD (the Somatization Behavioral Characteristics Questionnaire, SBCQ). Methods: N = 80 youth with SSD and 31 with non-SSD impairments participated in this study (age = 13.91 ± 2.72, 14 ± 3.21, respectively; females: n = 61, 14, respectively). Symptom intensity (Children’s Somatization Inventory-24; CSI-24), functional disability (Six-Minute Walk Test, walking rate of perceived exertion), and the SBCQ were assessed. SBCQ reliability and validity were examined. Results: SBCQ had acceptable reliability in both groups (Cronbach’s α > 0.7). Exploratory factor analysis in the SSD group revealed a three-cluster solution. Significant associations were found between the SBCQ, CSI-24, and functional disability. Both groups differed in the prevalence of all SBCQ behaviors. The greatest differences were in the mismatch between etiology and clinical presentation, and in the exhibited lack of trust in the therapist and “la belle indifference”. Receiver operating characteristic analysis showed that the SBCQ has moderate accuracy in discriminating between the two groups (area under the curve = 0.80). Sensitivity and specificity were 82.5% and 73.3%, respectively. Conclusions: The SBCQ is psychometrically sound. Findings may aid in developing sensitive assessment tools for SSD and continuing education for therapists.

## 1. Introduction

Somatic symptom disorder (SSD) is defined as the occurrence of distressing somatic symptoms along with abnormal thoughts, feelings, and behaviors in response to these symptoms [1]. These symptoms do not generally signify serious illness, although they represent atypical bodily sensations or discomfort. SSD accounts for as many as 50% of new medical outpatient visits in European countries [2], with female predominance [3]. Among the pediatric population, SSD places a heavy burden on families and on healthcare systems [4].

Within the large group of youth with SSD, a growing subgroup presents combined sensory symptoms, psychological symptoms, as well as neuro-functional motor problems (e.g., paralysis, weakness) [5]. Thus, the nature of these symptoms requires a multidisciplinary rehabilitation framework [3], including medical, psychiatric, and allied health professions (e.g., physical therapists) [5,6]. An experienced physiotherapist may easily recognize a child/youth with SSD due to their atypical motion characteristics, for example, inconsistencies among walking patterns and the nature of limb movement fluency. According to Bayesian theory [7], when a patient with Parkinson’s disease is asked to conduct a movement while focusing on reducing tremor in a limb, the tremor is reduced. In contrast, among SSD patients, the tremor is increased.

Youth with SSD may exhibit distinct behavioral characteristics, including avoidance behaviors and altered communication patterns with their clinical therapists. For instance, during physical rehabilitation sessions, individuals with SSD often display exaggerated symptoms and show a heightened sensitivity towards interpersonal interactions and expressions [8]. Moreover, anxiety and depression in this specific population are manifested differently, often through aggressive and agitated behaviors, which might also affect their ability to communicate effectively with therapists [9].

Another phenomenon that has recently gained more attention in the context of SSD is somatosensory amplification (SA). SA is defined as the tendency to experience typical body sensations as harmful, intense, and disturbing [10,11,12]. It comprises one’s disposition to focus on unpleasant sensations, which can be physiological and psychological, and to perceive them as abnormal. SA has three elements: (1) hyper-vigilance, which includes frequent self-scrutiny and heightened attention to unpleasant bodily sensations; (2) a tendency to focus on weak and infrequent sensations; and (3) a tendency to overestimate visceral and bodily sensations as aberrant and pathological instead of perceiving them as normal. These cognitive appraisals are associated with stress and anxiety and pose a threat to one’s conscious experience of the symptoms [10,11]. Although SA is involved in the general process of SSD and is significantly associated with it [12], youth with SSD might seem indifferent to their condition (“la belle indifference”) [13].

Given the aforementioned behavioral characteristics of youth with SSD, healthcare providers often describe these patients as “challenging” and “difficult to treat” [8]. These perceptions can significantly impact the therapeutic alliance and rapport, potentially resulting in an ambivalent response from the therapist towards the child and their family. It can also create difficulties in fostering a trusting and positively motivated therapeutic relationship, making it challenging to effectively engage the child in the therapeutic process. Therefore, achieving successful treatment outcomes necessitates a comprehensive understanding of the primary behavioral characteristics exhibited by youth with SSD. Understanding the unique behavioral characteristics of children with SSD might also aid in identifying these children and reducing unnecessary diagnostic procedures typically conducted prior to an SSD diagnosis. However, most of the characteristics described above were studied within the adult population with SSD but not within the pediatric population [13]. Therefore, a quick, accurate screening tool for SSD is critical for efficient treatment [14]. Furthermore, gaining insight into the typical behavioral characteristics associated with SSD can assist physical therapists in effectively treating these children who are often perceived as “challenging”. It can also provide valuable guidelines to support the therapeutic relationship and enhance the overall therapeutic process [15].

Therefore, the major aim of this study was to develop and to examine the validity and reliability of an observational measure of the behavioral characteristics of youth with SSD during physical rehabilitation. More specifically, the prevalence of the following 11 behaviors among children and youth with SSD were examined: behavioral lability, cooperation with therapist, analgesic resistance, resentment towards activity variations, lack of trust, “la belle indifference”, amplification of symptoms, gap between formal and informal evaluation, mismatch between etiology and clinical presentation, physiological markers of pain, and detailed description of painful events.

## 2. Materials and Methods

### 2.1. Participants

#### 2.1.1. Study Group—Children and Youth with SSD

Children and youth with SSD were referred to an ambulatory rehabilitation clinic in the center of the country by their primary/secondary care physician during the period 2017–2023. Children were admitted to the pediatric rehabilitation department after undergoing comprehensive medical tests with no clear findings of any physical, neurological, or orthopedic impairment.

Inclusion criteria: (1) age 8–18 years; (2) a diagnosis of SSD based on the Diagnostic and Statistical Manual of Mental Disorders (5th edition) (DSM-5; American Psychiatric Association, 2016) [1] criteria (300.82; ICD-F 45.1); (3) admittance to the ambulatory pediatric rehabilitation clinic; and (4) exhibition of gait disorders, lower limb weakness and/or paralysis or sensation disorder due to SSD. Exclusion criteria: (1) pain symptoms with no motor/sensory involvement; (2) exhibition of only upper-extremity disorders due to SSD; (3) hospitalized in the inpatient rehabilitation department; and (4) other clinical diagnoses, such as physical impairment and/or other psychiatric diseases.

A total of 91 children and adolescents with SSD were screened for eligibility. Six children were excluded due to inpatient hospitalization and five children were excluded because they presented only upper-extremity impairment; thus, a total of 80 children and youth with SSD participated in this study.

#### 2.1.2. Comparison Group—Children and Youth with Non-SSD Impairments in Body Functions and Structures

The comparison group comprised children and youth who had impairments in body functions and structures and were admitted to the ambulatory and inpatient pediatric rehabilitation clinic, but not due to SSD (e.g., acquired brain injury and orthopedic injuries). Children and youth were referred to the clinic by their primary/secondary care physician or by a different department in the hospital or other hospitals. Children were assessed during their first week at the pediatric rehabilitation department. Excluded from the study were children and youth (1) with an overt orthopedic condition (e.g., amputation or usage of a cast); (2) hospitalized in the inpatient rehabilitation department; (3) with other clinical diagnoses, such as other psychiatric diagnoses; and (4) presenting severe cognitive impairments that prevented them from being able to follow simple instructions.

### 2.2. Outcome Measures

According to what is recommended in the literature [16], the assessments were conducted using indirect (i.e., informal behavioral observations) [3,17] and direct measures. Indirect assessment involves observation by a physical therapist to assess the child’s functional capacity (e.g., posture, use of limbs, abnormal movements, and signaling of pain) while the child is engaged in a task/game. In a direct assessment, the therapist completed traditional objective measures of SSD severity and functional disability. Below we describe the indirect and direct measures used in the current study.

#### 2.2.1. Indirect Measures

During the initial 10–15 min of the indirect assessment, the therapist engaged in a conversation with the child, discussing their concerns and reasons for seeking rehabilitation. In the second part, the therapist played with the child using age-appropriate games (e.g., ball games, “four in a row”). At the end of the session, the therapist documented the child’s behavior and the dynamics between the therapist and child (including the level of engagement, cooperation, signs of fatigue, and distress exhibited by the child).

#### 2.2.2. Direct Measures

Demographics and medical information. Demographic and medical information (hospitalization duration, location of pain) were retrieved from the child’s medical records.

Somatization severity. Somatization symptom severity was evaluated using the Children’s Somatization Inventory-24 (CSI-24) [18]. The CSI-24 child self-report form was used. The questionnaire includes a list of 24 symptoms. Participants were asked to rate the intensity of each symptom within the past two weeks on a 5-point scale from “not at all” (0) to “a whole lot” (4). Higher scores (0–96) indicated a higher intensity of symptoms.

Functional disability. Functional disability was assessed using the Six-Minute Walk Test (6MWT) [19]. Participants were instructed to walk for 6 min as far as they could at a comfortable pace using their routinely used assistive aids/devices. At the end of the test, participants were asked to report their rate of perceived exertion (RPE) using the OMNI Walk/Run RPE (OMNI-RPE). The OMNI-RPE scale consists of a series of four pictures depicting a child walking up a hill and progressively appearing more tired, accompanied by corresponding descriptive words. The test has been validated for typically developing children aged 8 to 18 years [20,21,22].

Somatization behavioral characteristics questionnaire (SBCQ). Over the past decade, this research team has been providing treatment to children and youth with SSD in a pediatric rehabilitation setting. Drawing upon their extensive experience and a thorough review of the literature, this team has identified specific behaviors and reactions exhibited by children in response to the physical therapists’ assessments and interventions. Accordingly, they documented the behavioral characteristics of children/youth with SSD during direct and indirect evaluations. For example, the therapists carefully observed various aspects, such as the child’s mobility pattern upon entering the room, how the child described their condition, and their level of willingness to collaborate during the session. These observations provided valuable insights into the child’s behavior and reactions throughout the therapeutic process. Following these observations, a specific questionnaire was developed to assess the behavioral characteristics of youth with SSD. The questionnaire included 11 items scored as “yes” (1 point; the behavior characteristic was observed throughout the interaction with the child), “sometimes” (1/2 point; the behavior characteristic was occasionally observed during the interaction with the child), or “no” (0 points; the behavior characteristic was not observed during the interaction with the child). The scoring of items 4 (willing to make variations in the activity) and 10 (exhibits physiological markers of pain) was reversed. A total score was calculated by summing all item scores ranging from 0 to 11, with a higher score representing a higher representation of SSD behavior characteristics. Physical therapists were instructed to complete the questionnaire at the end of the second treatment session. Table 1 lists the items of the SBCQ.

### 2.3. Procedure

#### 2.3.1. SSD Group

Upon admission to the pediatric rehabilitation clinic, all participating children underwent a comprehensive assessment as part of a routine physical and psychological evaluation. Direct and indirect assessments were conducted during the first two physical therapy sessions.

Integrative pediatric rehabilitation program: Today, there is no standard approach to the treatment of SSD. However, accumulating evidence illustrates the feasibility and importance of treating SSD in an interdisciplinary setting [34]. Accordingly, the rehabilitation program in the current study encompassed a comprehensive approach, involving a multidisciplinary team consisting of physicians, physiotherapists, occupational therapists, individual educational lessons, weekly family–team meetings, and psychological therapy. The program consisted of both psychological therapy for the child and counseling sessions for the parents [35].

The program’s main goals were a return to age-appropriate functioning (such as independence in activities of daily living and school attendance), minimizing recurrence of symptoms (movement/walking impairment), and presenting a new model of parent–child communication. The program was conducted once or twice a week in an ambulatory format. For additional information about the integrative rehabilitation program administered (which was not the focus of the current study), please see Gerner et al. [3]

#### 2.3.2. Comparison Group—Children and Youth with Non-SSD Impairments in Body Functions and Structures

The comparison group also received the integrative pediatric rehabilitation program. The psychological interventions with children/youth and with their parents focused on: (1) the traumatic medical event; (2) the expected outcomes following the injury; and (3) psychoeducation and emotional support for the parents. The psychological interventions were provided once/twice per week. Physical therapy sessions commonly involved the following components: preventing secondary complications (e.g., contractures and weakness), fitness, and functional training (e.g., sit-to-stand training and gait training). Physical therapy was conducted at least twice/day, six days/week.

All study procedures were approved by the Sheba Medical Center Ethical Review Board (7394-20-SMC) and were conducted in accordance with the principles of the Helsinki Declaration and in line with the unified code of ethics of the American Psychological Association.

### 2.4. Statistical Analysis

#### 2.4.1. Descriptive Statistics and SBCQ Scores

Descriptive statistics (means, standard deviations, ranges, and percentages) were used to describe all study outcome measures. Differences in SBCQ scores between males and females were evaluated using the Mann–Whitney U test, and age associations with SBCQ scores were evaluated using Spearman’s correlations.

#### 2.4.2. Reliability Analysis of SBCQ

The internal consistency of the SBCQ was examined using Cronbach’s alpha (α) coefficient values [36]. The effect of dropping items was assessed by examining α change if an item was deleted. Overall reliability was assessed by the intraclass correlation coefficient (ICC). The value of the ICC was interpreted as follows: poor (<0.5), moderate (0.5–0.75), good (0.75–0.9), and excellent (>0.9) [37].

The SBCQ’s factor structure was examined using exploratory factor analysis (EFA). EFA was conducted using the principal component analysis (PCA) extraction method, followed by orthogonal (varimax) rotation to maximize variance. Before conducting PCA, various statistical assumptions necessary for PCA were tested [38]. The Kaiser–Meyer–Olkin (KMO) index of sampling adequacy was set at >0.75. Bartlett’s test of sphericity has to be highly significant (*p* < 0.001) [39]. In addition, multicollinearity was examined via the variance inflation factor (VIF). A VIF of >2.5 might indicate a multicollinearity problem [40]. The optimal number of factors was determined by latent root criteria (eigenvalues > 1.0, Kaiser’s criterion K1) and inspection of the scree plot [38,41]. Per factor, a criterion of three variables was set as the minimum, as a factor with fewer than three items is generally weak and unstable [42]. An item with a communality of less than 0.40 was removed from the analysis [41] and PCA was computed again. The “cross-loading” of items (i.e., an item that loads at 0.32 or higher on two or more factors) [42] was evaluated, and cross-loading items were dropped from the analysis. In addition, to assess the fit of the factor models, we examined the differences between the model-based correlations and the observed correlations. No more than 50% of the residuals should be greater than 0.05 [38]. Once no communalities, cross-loadings, or residual issues were identified, the PCA was completed.

#### 2.4.3. Convergent and Discriminative Validity of the SBCQ

Convergent validity was examined only in the SSD group via the evaluation of SBCQ associations (Pearson correlation) with the measure of somatization symptom severity (CSI-24) [18] and functional disability (6MWT and walking RPE). Discriminative validity was studied via the examination of differences between the two study groups in prevalence of each of the 11 SBCQ items using Chi-squared tests. Finally, the accuracy of the SBCQ total score in discriminating between youth with and without SSD was evaluated using receiver operating characteristic (ROC) curve analysis [43,44]. In a ROC curve, the true positive rate (sensitivity) is plotted in function of the false positive rate (100-specificity) for different cut-off points of a parameter. Each point on the ROC curve represents a sensitivity/specificity pair corresponding to a particular decision threshold. The area under the curve (AUC) is a measure of how well a parameter can distinguish between two diagnostic groups (diseased/normal). For the purpose of this study, the best cut-off value, positive predictive value, negative predictive value, and AUC were calculated. For the AUC of the ROC curve, 0.5 < AUC < 0.7 is less accurate, 0.7 < AUC < 0.9 is moderately accurate, 0.9 < AUC < 1.0 is very accurate, and AUC = 1.0 is perfectly accurate [45].

ROC analysis was conducted using the MedCalc 14.8.1 Statistical Program (MedCalc Software, Ostend, Belgium). All other analyses were carried out using the Statistical Package for the Social Sciences (SPSS) version 21.0 for Windows operating system (IBM Corporation, Armonk, NY, USA).

Statistical significance was set at 0.05 (two-tailed).

## 3. Results

### 3.1. Participant Demographic and Clinical Characteristics

The SSD and the non-SSD groups consisted of 80 and 31 children/youth, respectively. No statistically significant between-group age differences were observed (SSD group mean age: 13.91 + 2.72 years; non-SSD group: 14.00 + 3.21; t = 1.51, *p* = 0.13). However, in the SSD group, the prevalence of females was greater than that observed in the non-SSD group (76 and 45%, respectively). Additionally, no between-group differences were observed in the mean of duration hospitalization (SSD: 5.50 + 3.61; non-SD: 5.65 + 2.21; t = 0.23, *p* = 0.81) (see Table 2). No between-sex differences in both study groups were observed in the SBCQ scores (*p* > 0.05). No statistically significant associations were observed between age and SBCQ scores in the two study groups (*p* > 0.05).

### 3.2. SBCQ Reliability and Factor Analysis

Among both the SSD and the non-SSD groups, the SBCQ had acceptable reliability (Cronbach’s α = 0.76 and 0.82, respectively). Dropping variables analysis showed that no item decreased the questionnaire’s reliability (Table 3). The ICC of the questionnaire was excellent in the SSD group (ICC = 0.92, 95% confidence interval = 0.88–0.95) and good in the non-SSD group (ICC = 0.75, 95% confidence interval = l0.64–0.80).

In the SSD group, before conducting the EFA, we tested several of the statistical assumptions for such analyses. The KMO index was 0.825, and Bartlett’s test of sphericity was statistically significant (*p* < 0.0001). These results indicate that the sample size was adequate and that the extracted factors accounted for substantial observed variance. The SBCQ has 11 items. The initial examination of the items using EFA revealed that all item commonalities were acceptable (>0.40). Further, using varimax rotation, we observed no cross-loadings. Accordingly, all 11 items were included in the analysis. The K1-criterion and scree plot indicated a three-factor solution explaining 62.11% of the variance. The communalities ranged from 0.44 (item 4) to 0.78 (item 11). The first and second factors consisted of four items each, whereas the third factor consisted of three items (for factor loadings, see Table 4). Judged by the items’ content, the first factor was composed of items describing the child’s experience of pain; the second factor was composed of items that reflected behavioral incongruences; and the third factor included items describing the child’s engagement with the therapist and in the activity.

We were not able to conduct EFA for the non-SSD group as at least one of the variables had zero variance.

### 3.3. Convergent Validity

SBCQ total score statistically significantly correlated with somatization severity, as assessed with the CSI-24 (r = 0.53, *p* < 0.001), and with functional disability, as assessed with the 6MWT (r = −0.40, *p* < 0.001) and RPE during walking (r = 0.28, *p* = 0.02).

### 3.4. Discriminative Validity

Statistically significant between-group differences in the prevalence of behavioral characteristics were observed in all the behaviors examined in the SBCQ. The items with the greatest difference in prevalence (>60% difference in prevalence) were mismatch between etiology and clinical presentation (72.50% and 3.22% in the SSD and non-SSD groups, respectively), exhibits lack of trust in the therapist (65.00% and 0.00% in the SSD and non-SSD groups, respectively), and exhibits “la belle indifference” (65% and 3.22% in the SSD and non-SSD groups, respectively). For additional information, refer to Table 5.

When the various items comprising the SBCQ were summed, the total score of the two study groups varied considerably, with a mean score of 5.53 + 2.46 in the SSD group and 2.26 + 2.28 in the non-SSD group (t = 5.37, *p* < 0.001).

**Figure 1:**
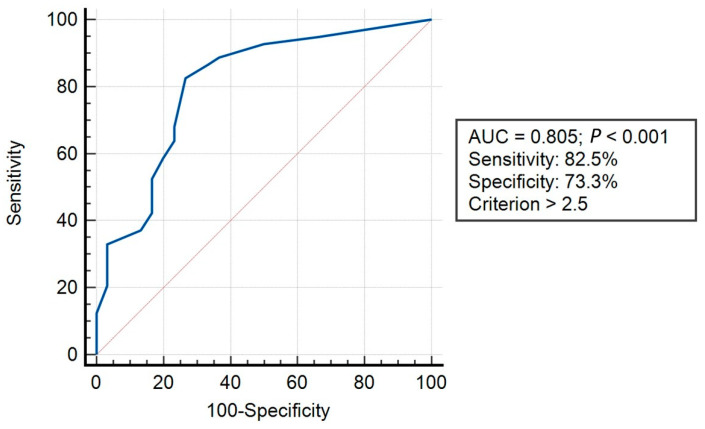
Receiver operating characteristic analysis. Notes: AUC, area under the curve; Blue line, represents the true positive rate (sensitivity) which is plotted in function of the false positive rate (100-specificity) for different cut-off points of a parameter. Each point on the curve represents a sensitivity/specificity pair corresponding to a particular decision threshold; Red line, represents area under the curve of 0.5.

In order to further analyze SBCQ’s ability to discriminate between youth with and without SSD, ROC analysis was conducted. The ROC showed that the SBCQ has moderate accuracy in discriminating between the two groups of youth with AUC of 0.80 (*p* < 0.001). The sensitivity and specificity of the newly developed SBCQ were 82.5% and 73.3%, respectively, with a criterion score of >2.5 (Figure 1).

## 4. Discussion

Pediatric SSD differs from that observed in the adult population, both in quality and quantity. However, most of the information about behavior and communication with healthcare providers among individuals with SSD is reported only among adults [13]. The unique behavioral characteristics exhibited by individuals with SSD can significantly impact the patient–client rapport and assist in identifying this at-risk population during the initial stages of physical therapy. Therefore, the major aim of this study was to develop and examine the reliability and validity of an observational measure of youth with SSD behavioral characteristics during rehabilitation.

### 4.1. SBCQ Demographic Characteristics—Sex and Age Differences

In both study groups, no statistically significant differences in SBCQ scores were observed between males and females. These results suggest similar behaviors during therapy among both males and females. These results were surprising as sex differences are often found in the symptoms or behaviors exhibited by children, even in cases with the same diagnosis. These sex differences are often consistent with general sex differences in healthy populations and may emerge in part due to more general socialization processes [46]. The prevalence of males in the SBCQ population in the current study is statistically significantly smaller than the prevalence of females. Therefore, to better understand sex differences in children and youth with SSD behavior during therapy, future studies with a higher prevalence of males are warranted. Similarly, no statistically significant association was found between age and SBCQ score. However, although the age range in the current study was wide (8.00–17.90 and 7.50–16.50 in SBCQ and control groups, respectively), most study participants in both groups were adolescents (mean age 13.91 ± 2.72 and 14.00 ± 3.21). Therefore, the generalizability of this study’s results to younger children is limited. However, a similar age mean is commonly reported in other SSD studies [3].

### 4.2. SBCQ Reliability

Overall, the SBCQ was found to be reliable, with acceptable reliability in both study groups (Cronbach’s alpha = 0.76 and 0.82 in the SSD and non-SSD groups, respectively). When comparing the reliability of the SBCQ to a commonly used measure of somatic symptoms, the CSI, the newly developed questionnaire had lower reliability. The lower reliability of the SBCQ may be related to its length, namely, only 11 items. More specifically, there is a consensus in the literature that internal reliability (the value of alpha coefficient) depends on the number of items. For example, the long version of the CSI, which consists of 35 items, has a good internal consistency, with Cronbach’s alpha = 0.90, compared to the shorter version, which has 24 items and a slightly smaller Cronbach’s alpha = 0.88 [18]. In other words, the higher the number of items, the higher the value of alpha will be [47]. However, the SBCQ was specifically designed for clinical settings, with a focus on being concise and practical in its application. Given the time constraints typically present in clinical settings and considering the questionnaire’s acceptable reliability and promising validity results, the necessity of adding additional items to improve its reliability becomes questionable.

### 4.3. SBCQ Convergent Validity

Total SBCQ score statistically significantly correlated with somatization severity (CSI-24) and functional disability level (6MWT). Considering that the CSI-24 is an SSD-specific outcome measure, the association observed is encouraging and contributes to the SBCQ validity. These results support the strong affinity between SSD intensity and child’s behavioral characteristics. Hence, the SBCQ can serve as a valuable tool for physical therapists, drawing attention to the potential diagnosis of SSD in children who have not yet been diagnosed. This holds particular significance in countries where individuals can receive physical therapy without a referral from a physician.

The association with functional disability, as assessed by the 6MWT, is also promising. The association between the two measures can be explained by studies that suggest that performance in the 6MWT may be influenced by motor cortex activity and that children with SSD have reduced activity in the motor-system-related brain areas, such as the gray matter in the primary motor cortex [48]. Taking into account findings in the literature on the brain function of children with SSD, the correlation between the SBCQ and the 6MWT is not unexpected and provides further support for the validity of the SBCQ.

Walking RPE was also associated with SBCQ. RPE is influenced by physiological factors, such as muscle fatigue [49,50,51]. However, subjective RPE scores are also thought to be influenced by affective or emotional qualities, such as anxiety [50,51,52]. In the current study, the two study groups had similar walking distances; however, in the SSD group, RPE was significantly higher (5.33 and 3.23 in the SSD and non-SSD group, respectively). These results may suggest that among youth with SSD, the affective, and not the physiological, component has the greater impact on RPE, in comparison to non-SSD youth. Similar results pertaining to RPE of youth with SSD were recently reported by Landa et al. [53]. Their study compared walking ability and RPE pre and post rehabilitation between adolescents with SSD and adolescents with traumatic brain injuries (TBIs). At pre-test, the TBI group presented a lower RPE than the SSD group (3.38 ± 2.49 and 6.25 ± 2.71, respectively), despite walking the same distance and being at a higher percentage of maximal heart rate.

### 4.4. Discriminative Validity

The prevalence of somatic behavioral characteristics was significantly different between the SSD and the non-SSD comparison group. The most salient behavioral characteristic was the mismatch between symptom etiology and clinical presentation (72% of the SSD sample), which hinders the ability to reach a clear diagnosis. For example, in our sample, a child with a significant lower-limb weakness (lower paraplegia) moved around independently in the department using a manual wheelchair. However, they could not use their upper extremities when asked to transfer from a wheelchair to a bed or to move from a supine to a prone position in bed. This finding aligns with previous reports from an adult SSD sample in which patients presented many symptoms that were difficult to cluster into a meaningful diagnosis [54].

Somatosensory amplification (i.e., exhibits intensification of the symptoms) was present in 62.5% of the children with SSD. For example, children described their pain as intensified by contact with water or air. This somatic amplification may be associated with increased autonomic responses associated with anxiety [55]. Another possible explanation for SA is that youth with SSD often arrive at healthcare centers following an extensive series of medical evaluations and assessments, with parents or even physicians responding to their pain or symptoms dismissively (e.g., “it’s all in your head”). Therefore, youth with SSD might not trust rehabilitation staff (65% of the SSD sample exhibited a lack of trust in the therapists) and might fear that their pain will be dismissed again. In doing so, they might feel urged to prove that it “really hurts” or “really prevents functioning”. On the other hand, youth might also amplify pain symptoms as a result of parental illness-reinforcing behaviors [18,56].

Another important finding was that 65% of the SSD sample exhibited “la belle indifference”. The frequency of this characteristic in SSD has been called into question, especially among children and youth. More specifically, to the best of our knowledge, only one study has addressed “la belle indifference” among children, reporting that only 24% of study participants presented with “la belle indifference” [13]. In contrast, we found a higher prevalence of “la belle indifference”. Differences between studies may be due to differences in SSD severity. More specifically, in the current study children also presented with limitations in social and/or academic function; in Samuels et al. [13], SSD severity level was less severe, as for most children (55%), the greatest disruption experienced was visiting the hospital for a diagnostic workup. “La belle indifference” goes along with presenting no physiological markers of pain (52% of the SSD sample). Interestingly, the combination of la belle indifference and no physiological markers of pain contradicts the finding that most children presented with SA. These results demonstrate that the complex nature of SSD may reflect a distinct aspect of the mind–body dissociation within the pediatric SSD population.

An additional strength of the current study was the indirect evaluation of the children (e.g., while entering the room), in addition to their direct evaluation using formal tests and measures. Surprisingly, in contrast to previous findings [16], we did not find significant differences in the prevalence of children presenting with a gap between direct and indirect evaluations. This may be due to the fact that the SBCQ was completed by therapists based on the first two therapy sessions, before an initial rapport between the child and the therapist was established.

In this study, we also used ROC analysis in order to further our understanding of the ability of the SBCQ to discriminate between youth with/without SSD. The analysis showed that the scale’s sensitivity level (82.5%) is higher than its specificity (73.3%). Sensitivity and specificity are inversely related: as sensitivity increases, specificity tends to decrease, and vice versa [57,58]. In addition, sensitivity and specificity are not fixed test characteristics, but test properties that describe the behavior of a test in a particular situation. As the setting, filter, or patient group changes, prevalence and accuracy may change [59]. The sensitivity of the SBCQ is overall good and comparable to the long version of the CSI (84%) [18]. These results indicate that the SBCQ has a good ability to distinguish between individuals with and without SSD.

This study has several limitations. First, the impact of various child (e.g., psychological factors such as anxiety) and parent background characteristics (e.g., socioeconomic status, educational level, and psychological status) was not evaluated. Second, study participants were children and youth with SSD admitted to an ambulatory pediatric rehabilitation clinic. Excluded from this study were those admitted to the inpatient clinic. Children and youth admitted to the inpatient clinic present with more severe symptoms than those admitted to the ambulatory clinic. Therefore, our results may be generalized only to patients with SSD admitted to ambulatory clinic. Third, our results were obtained by a well-trained team, which might limit the generalizability of the results to other physical therapy settings. Finally, future studies should further examine the reliability and validity of the questionnaire using a larger sample size. Considering the study’s limitations, future studies encompassing wider range of outcome measures, children treated in different clinical settings (both ambulatory and inpatient), and evaluators with various levels of experience treating children and youth with SSD are warranted.

## 5. Conclusions

The SBCQ is a reliable and valid measure developed specifically to assess the behavioral characteristics of youth with SSD in physical therapy. More specifically, SSD severity (CSI-24), walking ability (6MWT), and perception of walking intensity (RPE) were all related to the behavioral characteristics measured by the SBCQ. These results mark a starting point in our comprehension of the relationship between SSD behaviors and the severity of SSD. Furthermore, the SBCQ was able to capture one of the core underling mechanisms of SSD, that is, the mismatch between symptom etiology and their clinical presentation. Raising awareness of the specific behavioral characteristics of SSD in the pediatric population is central to the therapist–client rapport as well as the therapeutic process. Moreover, understanding the unique behavioral characteristics of children and youth with SSD may reduce unnecessary diagnostic procedures typically conducted prior to the diagnosis and aid the development of effective rehabilitation programs.

## Figures and Tables

**Table 1:** Somatic symptom disorder behavioral characteristics questionnaire: item descriptions.

Behavioral Characteristic	Description	Reference(s)
Behavioral lability	The child exhibits pronounced behavioral lability during the evaluation. For instance, within the session, the child may shift from anger to indifferent expressions or complain of a high pain level and then suddenly smiles at another child in the room.	[16,23,24]
Cooperation with therapist	The child is unwilling to partake in the physical activity to improve function or decrease pain.	[25,26,27]
Analgesic resistance	The child exhibits analgesic resistance and complains that the medications given to them are not useful.	[28]
Resentment towards activity variations	The child is unwilling to make variations in activity. This behavior may represent behavioral rigidity, a feature common to many psychopathologies.	[29]
Lack of trust	The child exhibits a lack of trust in their therapist. For instance, the child may use statements that imply distrust (e.g., “I said that I can’t do it; why do you have to test it?”).	[17,26]
“La belle indifference”	The child exhibits “la belle indifference”: an apparent lack of concern or distress shown by some patients toward their symptoms. It is often regarded as a typical characteristic of conversion symptoms/hysteria.	[13]
Amplification of symptoms	The child exhibits an intensification of the symptoms of either pain or dysfunction (e.g., mobility level and ability to conduct activities of daily living). The concept of symptom intensification may reflect an exaggeration of negative affect and illness states.	[16,27]
Gap between formal and informal evaluation	Inconsistencies between the child’s motor impairment (direct) and level of activity (indirect), as suggested by the Bayesian approach.	[7]
Mismatch between etiology and clinical presentation	Youth with somatic symptoms disorder commonly do not present the expected recovery pattern. For example, 6 weeks after an ankle sprain, the patient still complains of an inability to walk, stand, touch the affected foot, and wash it.	[30]
Physiological markers of pain	Child’s nonverbal pain expressions. For example, smiling may represent a negative marker of pain (i.e., no pain). However, grimacing and tears may represent a positive marker of pain.	[31]
Detailed description of painful event	The child provides detailed descriptions of the event responsible for the presenting problem/pain including the hour of the day, the weather on that day, the object responsible for the injury, etc.	[32,33]

**Table 2:** Demographic and clinical characteristics of study participants.

Characteristics	Somatic Symptom Disorder Group (*N* = 80)		Non-Somatic Symptom Disorder Group (*N* = 31)		Between-Group Differences: t (*p*-Value) OR Chi-Squared (*p*-Value)
	Mean (SD) OR *n* (%)	Range	Mean (SD) OR *n* (%)	Range	
Age, years: mean (SD)	13.91 (2.72)	8.00–17.90	14.00 (3.21)	7.50–16.50	1.51 (0.13)
Sex					
Females, *n* (%)	61.00 (76.2)	-	14.00 (45.16)	-	9.65 (0.001)
Males, *n* (%)	19.00 (23.7)	-	17.00 (54.83)	-	
Hospitalization duration, months: mean (SD)	5.50 (3.61)	0.50–10.00	5.65 (2.21)	1.00–11.00	0.23 (0.81)
Children’s Somatization Inventory—child, score: mean (SD)	28.29 (13.97)	2.00–59.00	-	-	-
Rate of perceived exertion, OMNI scale: mean (SD)	5.33 (2.66)	0.00–10.00	3.23 (1.26)	0.00–5.00	−4.31 (<0.001)
Six-minute walk test distance, meters: mean (SD)	280.12 (120.13)	0.00–700.00	282.21 (150.45)	50.12–600.25	0.07 (0.94)

Notes: SD, standard deviation.

**Table 3:** Somatic symptom disorder behavioral characteristics scale—reliability with standardized variables (Cronbach’s alpha).

		Somatic Symptom Disorder Group (*N* = 80)		Non-Somatic Symptom Disorder Group (*N* = 31)	
		Alpha	Alpha Change	Alpha	Alpha Change
Total Cronbach’s alpha	Total	0.76	-	0.82	-
	95% lower confidence limit	0.67	-	0.78	-
Effect of dropping variables	Q1	0.70	−0.02	0.81	−0.01
	Q2	0.71	−0.02	0.81	−0.01
	Q3	0.70	−0.02	0.81	−0.01
	Q4	0.76	0.02	0.80	−0.01
	Q5	0.71	−0.01	0.83	0.00
	Q6	0.74	0.00	0.81	−0.01
	Q7	0.66	−0.07	0.81	−0.01
	Q8	0.68	−0.04	0.83	0.00
	Q9	0.67	−0.06	0.82	0.00
	Q10	0.77	0.03	0.81	−0.01
	Q11	0.72	−0.01	0.82	−0.00

**Table 4:** Exploratory factor analysis of the Somatic Symptom Disorder Behavioral Characteristics Questionnaire—somatic symptom disorder group (*N* = 80).

Item Number and Description		Factor Loadings			
		Factor 1	Factor 2	Factor 3	Total Variance Explained
11	Provides detailed description of painful event	0.78			
7	Exhibits intensification of the symptoms	0.68			
10	Physiological markers of pain	0.62			
3	Exhibits analgesic resistance	0.52			
8	There is a gap between the formal and informal evaluation		0.68		
6	Exhibits “la belle indifference”		0.67		
9	Mismatch between etiology and clinical presentation		0.64		
1	Exhibits pronounced behavioral lability		0.63		
5	Exhibits lack of trust in the therapist			0.60	
2	Not willing to partake in the activity			0.55	
4	Willing to make variations in the activity			0.44	
Variance explained		27.26%	21.45%	13.39%	62.11%

**Table 5:** Children with somatic symptom disorder and orthopedic disability: behavioral characteristics.

Item Description	Response	SSD (*N* = 80): *n* (%)	Non-SSD (*N* = 31): *n* (%)	Within-Group Differences—SSD: Chi-Square (*p*-Value)	Within-Group Differences—Non-SSD: Chi-Square (*p*-Value)	Between-Group Differences: Chi-Square (*p*-Value)
Exhibits pronounced behavioral lability	Yes	31 (38.75) ^b^	2 (6.45) ^b,c^	31.52 (<0.01)	13.64 (<0.01)	10.99 (<0.01)
	Sometimes	8 (10.00) ^a,c^	14 (45.16) ^a^			17.09 (<0.01)
	No	41 (51.25) ^b^	15 (48.38) ^a^			0.07 (0.77)
Not willing to partake in the activity	Yes	49 (61.25) ^b,c^	2 (6.45) ^c^	35.70 (<0.01)	34.07 (<0.01)	26.99 (<0.01)
	Sometimes	19 (23.75) ^a^	4 (12.90) ^c^			1.67 (0.19)
	No	12 (15.00) ^a^	25 (80.64) ^a,b^			42.21 (<0.01)
Exhibits analgesic resistance	Yes	34 (42.50)	6 (19.35) ^b,c^	36.10 (<0.01)	40.66 (<0.01)	5.11 (0.02)
	No	44 (55.00)	25 (80.64) ^a,c^			5.83 (0.01)
	N/A	2 (2.50)	0 (0.00) ^a,b^			0.78 (0.37)
Willing to make variations in the activity	Yes	40 (50.00) ^b,c^	23 (74.19) ^b,c^	14.60 (0.01)	32.46 (<0.01)	5.19 (0.02)
	Sometimes	23 (28.75) ^a^	1 (3.22) ^a,c^			8.33 (<0.01)
	No	17 (21.25) ^a^	7 (22.58) ^a,b^			0.01 (0.90)
Exhibits lack of trust in the therapist	Yes	52 (65.00) ^b,c^	0 (0.00) ^c^	39.60 (<0.01)	61.00 (<0.01)	37.56 (<0.01)
	Sometimes	15 (18.75) ^a^	0 (0.00) ^c^			6.35 (0.01)
	No	13 (16.25) ^a^	31 (100.00) ^a,b^			65.39 (<0.01)
Exhibits “la belle indifference”	Yes	52 (65.00) ^b,c^	1 (3.22) ^b,c^	76.55 (<0.01)	32.46 (<0.01)	34.11 (<0.01)
	Sometimes	0 (0.00) ^a,c^	7 (22.58) ^a,c^			18.58 (<0.01)
	No	28 (35.00) ^a,b^	23 (74.19) ^a,b^			13.56 (<0.01)
Exhibits intensification of the symptoms	Yes	50 (62.50) ^b,c^	5 (16.12) ^c^	46.65 (<0.01)	37.24 (<0.01)	18.74 (<0.01)
	Sometimes	8 (10.00) ^a,c^	1 (3.22) ^c^			1.46 (0.22)
	No	22 (27.50) ^a,b^	25 (80.64) ^a,b^			25.56 (<0.01)
There is a gap between the formal and informal evaluation	Yes	40 (50.00) ^b^	0 (0.00) ^c^	53.00 (<0.01)	56.30 (<0.01)	24.01 (<0.01)
	Sometimes	0 (0.00) ^a,c^	1 (3.22) ^c^			2.39 (0.12)
	No	40 (50.00) ^b^	30 (96.77) ^a,b^			20.06 (<0.01)
Mismatch between etiology and clinical presentation	Yes	58 (72.50) ^b,c^	1 (3.22) ^c^	89.43 (<0.01)	46.39 (<0.01)	42.29 (<0.01)
	Sometimes	0 (0.00) ^a,c^	2 (6.45) ^c^			4.38 (0.02)
	No	22 (27.50) ^a,b^	28 (90.32) ^a,b^			35.56 (<0.01)
Physiological markers of pain	Yes	42 (52.50) ^b,c^	9 (29.03)	16.48 (<0.01)	0.55 (0.45)	4.72 (0.02)
	Sometimes	17 (21.25) ^a^	12 (38.70)			3.34 (0.06)
	No	21 (26.25) ^a^	10 (32.25)			0.39 (0.52)
Provides detailed description of painful event	Yes	51 (63.75) ^b,c^	11 (35.48) ^b,c^	73.11 (<0.01)	23.57 (<0.01)	7.01 (<0.01)
	Sometimes	0 (0.00) ^a,c^	1 (3.22) ^a,c^			2.39 (0.12)
	No	29 (36.25) ^a,b^	19 (61.29) ^a,b^			5.64 (0.01)

Notes: ^a^, statistically significantly different from “Yes” (*p* < 0.005; 2-tailed); ^b^, statistically significantly different from “Sometimes” (*p* < 0.005; 2-tailed); ^c^, statistically significantly different from “No” (*p* < 0.005; 2-tailed).

## Data Availability

Data will be available upon request.
